# Effects of Patterned Sound Deprivation on Short- and Long-Term Plasticity in the Rat Thalamocortical Auditory System* In Vivo*

**DOI:** 10.1155/2016/3407135

**Published:** 2016-01-04

**Authors:** Chloe N. Soutar, Laura G. Rosen, Simon G. Rodier, Hans C. Dringenberg

**Affiliations:** ^1^Department of Psychology, Queen's University, Kingston, ON, Canada K7L 3N6; ^2^Center for Neuroscience Studies, Queen's University, Kingston, ON, Canada K7L 3N6

## Abstract

Postnatal sensory experience plays a significant role in the maturation and synaptic stabilization of sensory cortices, such as the primary auditory cortex (A1). Here, we examined the effects of patterned sound deprivation (by rearing in continuous white noise, WN) during early postnatal life on short- and long-term plasticity of adult male rats using an* in vivo* preparation (urethane anesthesia). Relative to age-matched control animals reared under unaltered sound conditions, rats raised in WN (from postnatal day 5 to 50–60) showed greater levels of long-term potentiation (LTP) of field potentials in A1 induced by theta-burst stimulation (TBS) of the medial geniculate nucleus (MGN). In contrast, analyses of short-term plasticity using paired-pulse stimulation (interstimulus intervals of 25–1000 ms) did not reveal any significant effects of WN rearing. However, LTP induction resulted in a significant enhancement of paired-pulse depression (PPD) for both rearing conditions. We conclude that patterned sound deprivation during early postnatal life results in the maintenance of heightened, juvenile-like long-term plasticity (LTP) into adulthood. Further, the enhanced PPD following LTP induction provides novel evidence that presynaptic mechanisms contribute to thalamocortical LTP in A1 under* in vivo *conditions.

## 1. Introduction

It is now widely recognized that experience-dependent plasticity of sensory systems is greatest during brief, “critical/sensitive” periods of early postnatal life and markedly declines following the closure of these periods [1, 2]. For example, the tonotopic organization of the rodent primary auditory cortex (A1) undergoes rapid, experience-dependent maturation during the first two to three weeks following hearing onset (around postnatal day (PD) 10 in rats) [3]. During this period, the cortical region responsive to tonal stimuli contracts and the juvenile overrepresentation of high frequencies is converted to a mature, more balanced frequency map [4]. Further, during the sensitive period, passive exposure to frequency-specific, pulsed tones results in competitive overrepresentation of those frequencies in A1, with the most dramatic changes occurring around PD 11–13 [3, 4]. The observation that a single, brief (8 to 25 min) exposure to broadband noise on PD 14 can impair the ability of rats to perform acoustic frequency discrimination in adulthood further emphasizes that there are important functional consequences of exposure to specific sounds during this period of auditory development [5].

Interestingly, sensory experience, or the lack thereof, can itself alter the duration and closure of sensitive periods of cortical development. Rats deprived of patterned acoustic inputs (by rearing under continuous white noise (WN) to mask patterned sound) show arrested cortical development, leaving A1 tonotopy in an immature, juvenile-like state [6]. The importance of patterned sensory stimulation for cortical maturation is also evident in assays that directly assess levels of synaptic plasticity in the thalamocortical auditory system. Long-term potentiation (LTP) of field potentials in A1* in vivo *(elicited by stimulation of the medial geniculate nucleus, MGN) is readily induced in juvenile rats (PD 30–50) but shows a sharp decline in adult animals, indicative of progressive synaptic stabilization over postnatal development [7]. Rats reared in continuous WN do not show this developmental reduction of plasticity, with LTP remaining at high, juvenile-like levels into adulthood [8, 9]. Together, this work indicates that the experience of patterned acoustic inputs is required for the appropriate development of tonotopy and stabilization of A1 synapses.

While effects on long-term plasticity have been examined, no work to date has characterized the impact of patterned sound deprivation on short-term plasticity in the thalamocortical auditory system. Understanding the characteristics and mechanisms of short-term plasticity is of particular importance in the auditory cortex, as acoustic communication (e.g., birdsong, rodent vocalizations, and human speech) often requires the processing of brief and repetitive sounds. For example, many rodent vocalizations (pup isolation calls, adult encounter calls) are repeated at rates between 3 and 10 Hz [10], and the ability to rapidly amplify (potentiate) or suppress neural responses to these repetitive inputs may play an important role in guiding behavioral responses to acoustic signals emitted by conspecifics. Two commonly studied forms of short-term plasticity are paired-pulse facilitation (PPF) and paired-pulse depression (PPD). The tendency of postsynaptic cells to exhibit either facilitation or depression of synaptic responses to rapid, successive inputs is a sensitive index of the developmental state and strength of synaptic communication [11]. Generally, mature synapses that are characterized by strong neuronal coupling and a higher probability of transmitter release exhibit PPD, whereas immature synapses with lower release probability tend to exhibit PPF with successive stimulation pulses [11, 12]. Therefore, synaptic responses to paired-pulse stimulation provide a sensitive measure of synaptic development and maturity.

In addition, paired-pulse responses are commonly used to assess the relative contributions of pre- versus postsynaptic mechanisms of LTP induction and expression. Previous work has shown that greater levels of PPD following LTP induction reflect presynaptic modifications, particularly increases in transmitter release probability or magnitude [13–17]. Most studies examining changes in PPF or PPD in relation to LTP induction have been conducted in the hippocampal formation, and it is unclear to what extent pre- and postsynaptic mechanisms contribute to LTP in thalamocortical sensory pathways.

With the present experiments, we assessed the effects of continuous WN rearing on both long-term plasticity (LTP) and short-term plasticity (PPF/PPD) in the thalamocortical auditory system of adult rats. Further, we analyzed paired-pulse responses before and after LTP induction, in order to further elucidate mechanisms of LTP in the thalamocortical auditory system* in vivo*.

## 2. Materials and Methods

### 2.1. Animals

All experiments were conducted in accordance with guidelines established by the Canadian Council on Animal Care and approved by Queen's University Animal Care Committee. All efforts were made in order to minimize animal suffering and the number of animals employed for these experiments. Pregnant (~19 days) female Long-Evans rats were obtained from Charles River Laboratories Inc. (St. Constant, Québec, Canada) and housed individually in a colony room (12 : 12-hour reverse light cycle, lights on at 19:00) with food and water available* ad libitum*. Pups were housed with their mother until weaning at PD 21, at which time males were selected and housed in groups of three to five.

### 2.2. Continuous White Noise Rearing

Pregnant rats were housed in sound attenuated chambers (114 × 61 × 66 cm, aluminum-lined plywood) maintained at standard colony room conditions. Each chamber was fitted with a time-controlled light, fan, and two equally spaced, ceiling-mounted speaker boxes. Each speaker box contained one 8-inch woofer and one 3.25-inch tweeter with frequency ranges of 45 Hz to 5 kHz and 2 to 35 kHz, respectively (American Legacy Series 2 Speakers, Legacy Audio, NY, USA). The speakers were connected to a custom-made WN generator (Technical Workshop, Department of Psychology, Queen's University). Prior spectral analysis showed that the WN signal covers a frequency range of up to ~35 kHz, with power gradually declining between 30 and 37.5 kHz [9]. Sound attenuation across the chamber wall was ~27 dB sound pressure level (SPL) for measurements taken immediately outside a chamber containing an ~80 dB SPL signal.

Exposure to WN began at PD 5, approximately five days before the onset of low-threshold hearing in rats [3], and was increased incrementally from ~65 to ~80 dB SPL over five days to limit stress experienced by the mother. The volume was subsequently maintained at ~80 dB SPL until PD 50–60. Control rats were housed in a sound attenuated chamber with the WN generator turned off until PD 50–60. At PD 50–60, electrophysiological procedures were conducted. Continuous WN rearing of rats using similar parameters has been shown to delay A1 tonotopic refinement [6] and alter properties of LTP in A1* in vivo *[9].

### 2.3. Surgical Preparation

Rats were removed from the sound chamber and deeply anaesthetized with urethane (Sigma-Aldrich, Oakville, Ontario, Canada; 1.5 g/kg administered intraperitoneally (i.p.) as three 0.5 g/kg doses, one every 15 min, with supplements as necessary). Following anesthesia induction, rats were mounted in a stereotaxic apparatus and the local analgesic bupivacaine (Hospira Healthcare Corporation, Montreal, Quebec, Canada; 5 mg/kg administered subcutaneously, s.c.) was applied to the scalp 15 min prior to the start of the surgery. Throughout the experiment, body temperature was monitored and maintained at 36-37°C.

An incision was made to expose the skull and burr holes were drilled over the medial geniculate nucleus (MGN, 5.5 mm posterior to bregma, 4.0 mm lateral to midline) and the ipsilateral A1 (4.5 mm posterior to bregma, 7.0 mm lateral to midline). Two additional holes were drilled in the contralateral parietal and frontal bones to secure ground and reference connections.

### 2.4. Electrophysiology

A concentric bipolar stimulation electrode (SNE-100, Rhodes Medical Instruments, David Kopf, Tujunga, CA, USA) was lowered into the MGN (5.4 to 6.4 mm ventral to the skull surface) to provide MGN stimulation (single 0.2 ms pulses). The stimulation electrode was connected to a stimulus isolation unit (ML180 Stimulus Isolator; AD Instruments, Toronto, Ontario, Canada) providing a constant current output. A monopolar recording electrode (125 *μ*m diameter Teflon-insulated stainless steel wire) was lowered into A1, aiming for the middle cortical layers (3.2 to 5.4 mm ventral to the skull surface). The final ventral depth of both electrodes was adjusted to yield maximal field postsynaptic potential (fPSP) amplitudes in response to single-pulse MGN stimulation. The recording electrode was connected to an amplifier (Model 1800, A-M Systems Inc., Carlsborg, WA, USA; half-amplitude filter settings at 0.3 Hz to 1 kHz) and A-D converter (PowerLab/4s system, Scope software v. 4.0.2, AD Instruments) that digitized (10 kHz) and stored the recorded signal for offline analyses.

### 2.5. Data Collection

Following electrode placement, the brain was allowed to stabilize for 30–45 min. The MGN was then stimulated at increasing intensities (0.1–1.0 mA in 0.1 mA increments) to generate an input-output series. The stimulation intensity yielding 50–60% of the maximal fPSP amplitude in A1 was used for the remainder of the experiment. For LTP experiments, fPSPs (one fPSP every 30 s) were recorded until 30 min of stable baseline responses was obtained (fPSP amplitudes falling within 5% of the average baseline fPSP amplitude). Subsequently, theta-burst stimulation (TBS) was applied to the MGN as four repeated trains, with each train consisting of 10 pulse bursts (bursts repeated at 5 Hz, each burst containing 5 pulses repeated at 100 Hz). Trains were repeated once every 10 seconds for a total of 40 bursts. After TBS delivery, recordings of fPSPs resumed for 60 min, followed by a second and third round of TBS, each of which was followed by 60-minute fPSP recording period.

To analyze short-term plasticity, paired-pulse stimulation was applied to the MGN at two time points: (a) immediately prior to the onset of baseline fPSP recordings and (b) at the end of the LTP experiment (i.e., 60 min after delivery of the third round of TBS). Two successive single pulses were delivered, with pulses separated by the following interstimulus intervals (ISIs): 25, 50, 75, 100, 125, 250, 500, and 1000 ms. Ten episodes of paired-pulse stimulation were delivered for each ISI, each separated by a 5000 ms interval.

### 2.6. Histology

Following the experiments, rats were perfused through the heart with 0.9% saline, followed by 10% formalin. Brains were removed and stored in 10% formalin for a minimum of 24 hours before sectioning coronally (40 *μ*m sections) using a cryostat. Slices were mounted onto microscope slides and electrode placements were verified using standard histological techniques. Data from experiments with inaccurate placements were discarded.

### 2.7. Data Analysis

**Figure 1:**
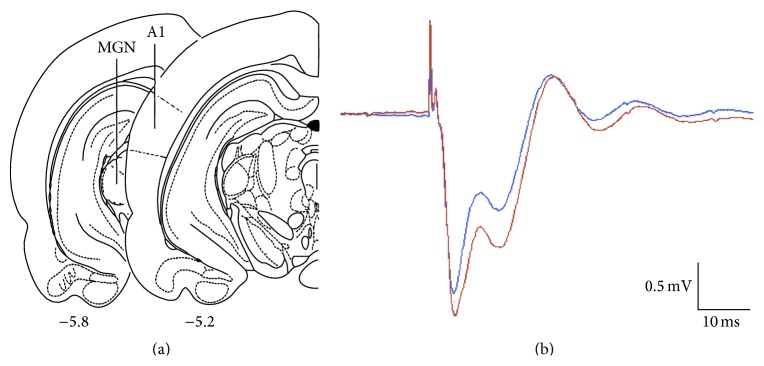
(a) Typical placements of the stimulating and recording electrodes in the MGN and A1, respectively. Atlas images adapted from Paxinos and Watson [18]. (b) Typical fPSPs recorded in A1 following single-pulse MGN stimulation consisted of two distinct negative peaks occurring at approximately 7 and 15 ms after the stimulation artifact (initial sharp, positive spike). Blue (smaller amplitude) and red (larger amplitude) traces represent fPSPs recorded before and after LTP induction, respectively.

All fPSPs were stored and analyzed using Scope software (v. 3.6.5, AD Instruments). In agreement with prior work [7, 8], fPSPs in A1 elicited by MGN stimulation consisted of two negative-going peaks (see Figure 1(b)). The amplitude of each peak was computed offline by calculating the voltage difference between the activity measured immediately prior to the stimulus artifact and that of the maximum peak negativity. Amplitude values were averaged over 10-minute intervals and normalized by dividing them by the average baseline amplitude of each animal.

For paired-pulse responses, fPSPs were averaged for each ISI, before and after LTP induction (pre-LTP and post-LTP, resp.). A paired-pulse ratio (PPR) was calculated for each rat at each ISI by dividing the peak amplitude (computed as described above) of the second fPSP by that of the first fPSP (note that PPRs were calculated only for the first of the two negative peaks of the fPSP). A PPR value of greater than 1.0 reflects PPF, whereas a PPR value of less than 1.0 reflects PPD of synaptic transmission.

All data are expressed as mean ± standard error of the mean (SEM). Statistical comparisons were made using mixed-model analyses of variance (ANOVA) and, where statistically appropriate, pairwise comparisons using the SPSS software package (version 21.0, SPSS Inc., IL, USA).

## 3. Results

### 3.1. LTP Experiments

The effects of WN rearing on long-term plasticity in the thalamocortical auditory system of adult rats were examined using LTP induction* in vivo*.  Figure 1(a) illustrates typical placements of the stimulation and recording electrode in the MGN and the middle layers of A1, respectively. In accordance with previous work using the same electrode configuration [7, 8, 19], single-pulse MGN stimulation elicited fPSPs in A1 consisting of two distinct, negative-going components, with latencies to peak of approximately 5–8 and 13–16 ms (Figure 1(b); blue (smaller amplitude) and red (larger amplitude) traces indicate typical fPSPs recorded before and after LTP induction, resp.). Previous current-source density analyses and pharmacological manipulations have demonstrated that the first and second negative peaks reflect sequential current sinks that correspond to initial activation of thalamocortical synapses (largely layer IV) and subsequent activation of intracortical synapses (around layers II/III), respectively [8, 19, 20].

Prior to the induction of LTP by TBS of the MGN, 60 fPSPs elicited by single-pulse MGN stimulation were recorded to obtain a stable measure of baseline synaptic strength. Stimulation pulse intensities (the current intensity that elicited 50–60% of maximal fPSP amplitude during input-output testing) used for the two experimental groups did not differ, with mean intensities of 0.361 ± 0.028 mA and 0.359 ± 0.027 mA for WN (*n* = 18) and age-matched control (*n* = 18) rats, respectively, *P* > 0.05. Comparisons of the amplitude of baseline (pre-TBS) fPSPs showed that WN-reared rats displayed smaller amplitudes compared to control animals. For the first fPSP peak, WN-reared and control rats exhibited mean peak amplitudes of 1.83 ± 0.29 mV and 2.64 ± 0.35 mV, respectively; amplitudes for the second fPSP peak in WN-reared and control rats were 0.82 ± 0.09 mV and 1.03 ± 0.09 mV, respectively. However, neither of these differences reached statistical significance, *P* > 0.05 for both comparisons (data not shown).

**Figure 2:**
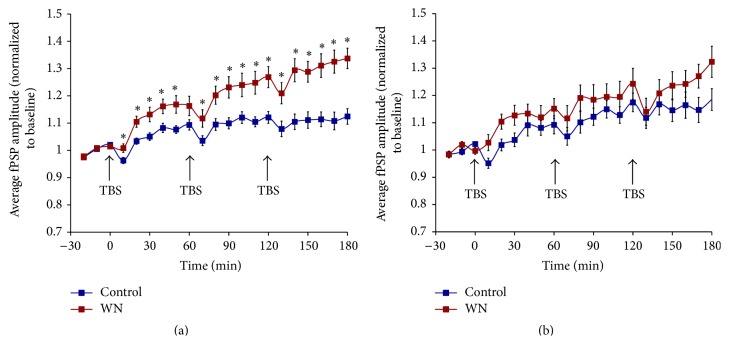
Amplitude of the first (a) and second (b) peak of fPSPs recorded in A1 of rats reared under WN (*n* = 18) and age-matched controls (*n* = 18) following MGN stimulation. TBS (indicated by arrows) of the MGN resulted in significant increases in the amplitude of both fPSP peaks in the two groups of animals. (a) For the first fPSP peak, rats reared in WN showed significantly more LTP than controls. ANOVA results for (a): effect of time,* F*(3.57, 121.25) = 43.9, *P* < 0.001; effect of rearing condition,* F*(1, 34) = 12.2, *P* < 0.05; effect of time by condition interaction,* F*(3.57, 121.25) = 7.9, *P* < 0.001; *∗* indicates significant simple effects, *P* < 0.05. (b) Rearing under WN did not significantly enhance LTP of the second fPSP peak. ANOVA results for (b): effect of time,* F*(4.88, 165.88) = 21.4, *P* < 0.001; no effect of condition,* F*(1, 34) = 2.0, *P* > 0.05; no effect of time by condition interaction,* F*(4.88, 165.88) = 1.3, *P* > 0.05.

Delivery of TBS to the MGN (total of three TBS episodes, delivered every 60 min; see Figure 2) resulted in reliable LTP induction in rats reared under WN and age-matched control animals. During the final 30 min of recording, rats reared under WN exhibited fPSP amplitudes of 132% and 128% of baseline for the first and second fPSP peak, respectively, whereas fPSP amplitudes in controls increased to 112% (first peak) and 117% (second peak; Figure 2). Statistical analyses (see Figure 2 caption for details) indicated that, relative to control animals, WN-reared rats showed significantly greater LTP for the first, but not the second, fPSP peak. The effect of WN rearing to enhance LTP was already apparent after the first TBS episode, with potentiation of the first fPSP peak reaching 116% and 109% in WM and control rats, respectively, and potentiation of the second fPSP peak reaching 115% and 109% in WN and control rats, respectively.

Together, these observations suggest that deprivation of patterned acoustic inputs during early postnatal life results in the maintenance of higher levels of long-term plasticity in A1 of rats into adulthood, an effect that is particularly pronounced for thalamocortical synapses (first fPSP peak; see above).

### 3.2. Paired-Pulse Analyses

**Figure 3:**
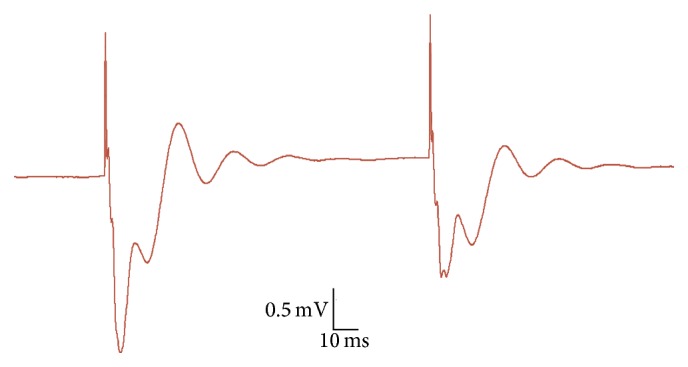
Typical fPSPs recorded in A1 following paired-pulse MGN stimulation. The trace depicted was elicited with an ISI of 100 ms and illustrates an example of PPD, with the amplitude of the second fPSP (first peak) suppressed relative to the first fPSP.

A subset of rats (*n* = 9 for both the WN and control condition) used for the LTP procedures was also subjected to the paired-pulse stimulating protocol. The paired-pulse analyses were limited to the first fPSP peak, given that only monosynaptic (in our case, thalamocortical) responses allow a direct assessment of presynaptic contributions to LTP induction [14, 15]. Paired-pulse responses (with ISIs ranging from 25 to 1000 ms) were measured both immediately prior to recording baseline fPSPs (pre-LTP) and 60 min after delivery of the final (third) TBS episode (post-LTP).  Figure 3 illustrates a typical recording of two successive fPSPs in A1 elicited by paired-pulse (100 ms ISI) MGN stimulation.

**Figure 4:**
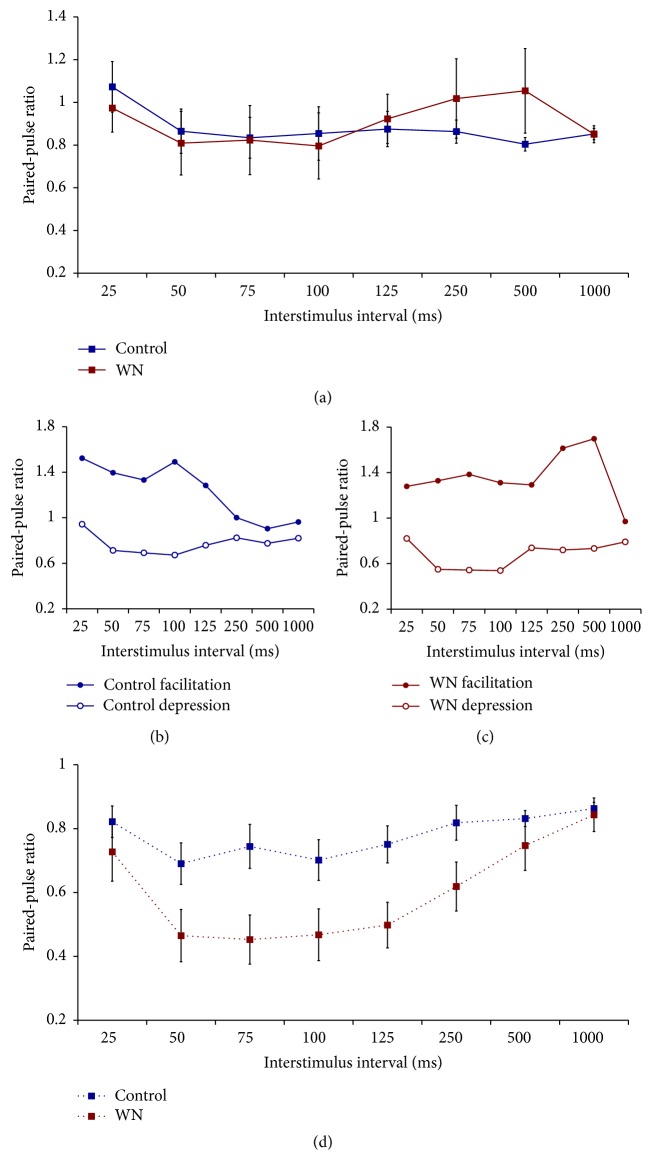
The effect of WN rearing on paired-pulse responses before (a, b, c) and after (d) LTP induction. (a) There was no significant effect of rearing condition on paired-pulse responses before LTP induction. As groups, both WN and control rats showed little evidence of PPD or PPF. ANOVA results for (a): effect of ISI,* F*(2.43, 38.88) = 1.78, *P* > 0.05; effect of condition,* F*(1, 16) = 0.04, *P* > 0.05; effect of ISI by condition interaction,* F*(2.43, 38.88) = 1.29, *P* > 0.05. (b) However, detailed analyses of responses in individual rats revealed that the majority (*n* = 7, control depression) of control rats exhibited PPD, with the remaining rats (*n* = 2, control facilitation) showing substantial PPF, which largely cancelled out the depression seen in the majority of control rats. (c) Similarly, most WN rats (*n* = 6, WN depression) showed clear PPD, and only a minority (*n* = 3, WN facilitation) exhibited PPF (note that panel (a) depicts the group means of data plotted in (b) and (c)). (d) Following LTP induction, both groups exhibited clear PPD. Further, WN-reared rats showed greater levels of PPD than those seen in control animals. ANOVA results for (b): effect of ISI,* F*(3.05, 48.84) = 10.57, *P* < 0.001; effect of condition,* F*(1, 16) = 6.21, *P* < 0.05; effect of ISI by condition interaction,* F*(3.05, 48.84) = 2.13, *P* > 0.05.

Prior to LTP induction, the group averages for both WN-reared and control rats showed very little evidence of either PPF or PPD across the entire range of ISIs (all paired-pulse ratios around 1.0 for both groups; Figure 4(a)). However, closer examination of the data showed that the majority of rats in both conditions (7/9 controls and 6/9 WN rats) showed clear PPD, in particular for ISI between 50 and 100 ms, while the remaining rats displayed relatively high levels of PPF (Figures 4(b) and 4(c)).

Interestingly, analyses of paired-pulse responses after LTP induction indicated that both groups of animals now exhibited clear PPD, an effect that was most pronounced for ISI between 50 and 125 ms (Figure 4(d)). In fact, following LTP induction, only one animal (control condition) showed slight PPF, with all other rats exhibiting clear PPD. Further, WN rats exhibited greater levels of PPD than those seen in control rats (Figure 4(d)). These data suggest that thalamocortical LTP induction is accompanied by a shift toward greater levels of PPD, an effect that is particularly pronounced for WN-reared animals.

**Figure 5:**
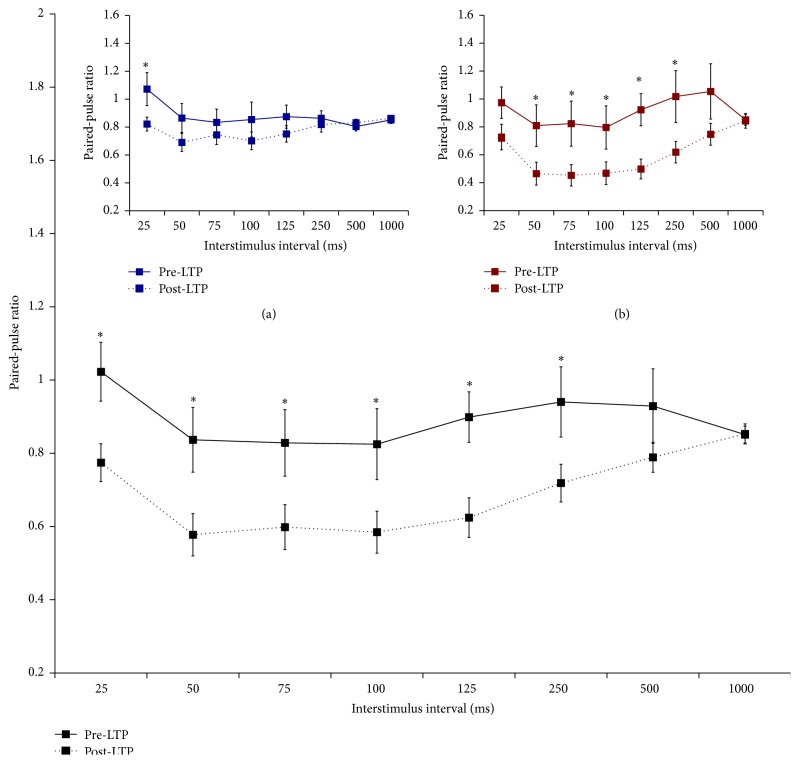
The effect of LTP induction on paired-pulse responses; data are collapsed across rearing conditions. LTP induction resulted in significant enhancement of PPD at ISIs of 25 to 250 ms. ANOVA results: effect of ISI,* F*(2.34, 39.85) = 4.16, *P* < 0.05; effect of LTP induction,* F*(1.00, 17.00) = 11.11, *P* < 0.05; effect of ISI by LTP interaction,* F*(3.38, 57.48) = 4.14, *P* < 0.05. Inserts (a) and (b) show the effect of LTP for control and WN-reared rats, respectively; *∗* indicates significant simple effects, *P* < 0.05.

The observation that, for both control and WN-reared rats, LTD induction was accompanied by a shift toward greater PPD is intriguing, given that changes in paired-pulse responses are taken as evidence for the involvement of presynaptic mechanisms in LTP (see above). Thus, to further explore this question, we performed a supplementary analysis on the effect of LTP induction on paired-pulse responses, collapsing across the two rearing conditions (*n* = 18). Prior to LTP induction, paired-pulse stimulation resulted in modest PPD, particularly for ISIs between 50 and 100 ms (Figure 5). Following LTP induction, PPD was significantly enhanced for ISIs ranging from 25 to 250 ms (Figure 5). Thus, the induction of LTP in the thalamocortical auditory system* in vivo *is associated with a clear enhancement of PPD during thalamic paired-pulse stimulation, an effect strongly suggestive of involvement of presynaptic mechanisms in LTP at thalamocortical synapses in A1 under the present experimental conditions.

## 4. Discussion

### 4.1. Summary of Results

In the present experiments, we examined the effects of depriving rats of patterned acoustic stimulation by continuous WN rearing (PD 5 to PD 50–60) on long- and short-term plasticity in the thalamocortical auditory system. Consistent with our previous findings [8, 9], WN rearing markedly altered long-term plasticity, as evidenced by the increase in LTP of WN rats compared to age-matched control animals raised in unaltered acoustic conditions. Surprisingly, short-term plasticity (PPF and PPD) was largely unaffected by WN exposure. However, LTP induction resulted in an enhancement of PPD, an effect that was particularly pronounced for WN animals. These data suggest involvement of presynaptic mechanisms in* in vivo *LTP induction in the thalamocortical auditory system of adult rats. 

### 4.2. WN Exposure Facilitates LTP

In agreement with previous work, single-pulse stimulation of the MGN reliably elicited fPSPs in A1, consisting of two negative-going components, with latencies to peak of about 5–8 and 13–16 ms, respectively. Prior work has shown that both peaks are strongly attenuated by local A1 application of the AMPA receptor antagonist CNQX [7], indicating that fPSPs elicited under the present experimental conditions largely reflect excitatory currents caused by cortical AMPA receptor activation. Importantly, current-source density analysis and pharmacological approaches have revealed that the first and second fPSP peaks reflect current sinks associated with thalamocortical (largely layer IV) and intracortical (layers II/III) synapses, respectively [8, 19]. Thus, the recording techniques employed in the present study allow for the concurrent assessment of plasticity at different levels of processing in the rodent A1.

The current experiments showed that delivery of TBS to the MGN resulted in reliable LTP induction for both fPSP peaks. The fact that both thalamocortical and intracortical synapses can exhibit potentiation (present results and [7, 8]) supports recent work suggesting that both sets of synapses can maintain plasticity into adulthood and could play a role in receptive field plasticity in both juvenile and mature animals [21]. Further, WN-reared rats exhibited greater levels of LTP compared to control animals, an effect that was apparent for both fPSP peaks, but reached statistical significance only for the first fPSP peak. Thus, thalamocortical synapses may be more sensitive to the lack of patterned acoustic stimulation during early postnatal life relative to intracortical synapses. However, previous work has shown that LTP of both fPSP peaks is significantly enhanced by chronic WN rearing [8, 19]; the reasons for this discrepancy (for the second fPSP peak) are not clear. Nevertheless, our results confirm that the absence of patterned sound during early postnatal life leaves A1 in a state of heightened plasticity, characteristic of immature, more malleable synaptic connectivity [1, 3, 4, 7, 22–25].

Prior work has established that levels of LTP provide a sensitive measure of heightened plasticity during critical/sensitive periods of cortical development. For instance, in the primary visual cortex* in vitro*, LTP can be readily induced during, but not after, the critical period for ocular dominance plasticity [26–28]. Similarly, in A1, juvenile rats (PD 30 to 35) exhibit substantially more LTP than young adults (PD 100), and little or no LTP is induced in rats older than PD 200 [7]. Thus, the present findings showing greater (juvenile-like) levels of LTP following WN rearing are consistent with the interpretation that patterned sound deprivation disrupts synaptic maturation in the rodent thalamocortical auditory system.

### 4.3. Effect of WN and LTP Induction on Short-Term Plasticity

The main novel contribution of the present experiments lies in the analysis of short-term plasticity and its relation to LTP in the thalamocortical auditory system* in vivo*. Prior to LTP induction, control animals collectively showed relatively little evidence for either PPF or PPD (PPR close to 1.0). However, closer examination of the data revealed that, in fact, the majority (7/9) of control animals showed clear PPD and that this effect was largely cancelled out by two animals exhibiting considerable PPF. This pattern of paired-pulse responses was not significantly altered in rats reared under WN conditions, with 6/9 rats showing PPD and the remaining 3 rats exhibiting PPF. The results indicate that, in clear contrast to the LTP findings, patterned sound deprivation does not result in significant alternations in short-term plasticity expression at thalamocortical auditory synapses.

The direction and magnitude of paired-pulse responses are, at least in part, related to the probability of transmitter release from the presynaptic terminal; higher release probability depletes the readily available pool of synaptic vesicles, resulting in reduced transmitter release and PPD at short ISIs [11, 12, 29, 30]. However, additional mechanisms also appear to play important roles in PPD. Specifically, recent work has shown that a reduction in calcium influx into the presynaptic terminal with repetitive stimulation can account for PPD at synapses of the hippocampus and the calyx of Held [31, 32]. Thus, care must be taken when interpreting changes in PPD, given that there are several candidate mechanisms that could mediate such changes of short-term plasticity at central synapses.

The observation that WN rearing did not significantly alter paired-pulse responses prior to LTP induction implies that patterned acoustic stimulation may not play a dominant role in the maturation of the presynaptic component of the thalamocortical auditory pathway, at least with regard to mechanisms involved in transmitter release or presynaptic calcium channels (see above). Also, the fact that WN rearing enhanced LTP without significantly altering paired-pulse responses suggests that alterations in these presynaptic components are not critically involved in the LTP facilitation induced by patterned sound deprivation. In support of this contention, prior work has identified postsynaptic mechanisms of LTP facilitation in WN-reared animals, particularly the upregulation of NMDA-GluN2B receptor subunits in A1 neurons into adulthood [33]. The observation that pharmacological blockade of GluN2B subunits in A1 reverses the LTP facilitation observed in WN-reared rats [8] provides strong support for the link between elevated GluN2B expression and heightened plasticity in acoustically deprived rodents.

We also analyzed the effects of LTP induction on paired-pulse responses in order to examine the relative contributions of pre- and postsynaptic mechanisms to LTP in the thalamocortical auditory pathway* in vivo*. LTP induction resulted in a significant enhancement of PPD in all animals, even though this effect was particularly pronounced in WN-reared rats. Since an enhancement of PPD is typically assumed to indicate increased transmitter release [[13–16]; but see the discussion above], our results indicate that LTP between MGN and A1* in vivo *is mediated, at least in part, by presynaptic, transmitter release-related mechanisms. Work by Hirata and Castro-Alamancos [34] has shown that long-term enhancement of field potentials recorded in the barrel cortex of anesthetized rats can be elicited by thalamic disinhibition, further supporting the involvement of presynaptic mechanisms in thalamocortical LTP.

### 4.4. Conclusion

In summary, the present study indicates that patterned acoustic deprivation during early postnatal development leaves the thalamocortical auditory system in a state of heightened plasticity. The failure of WN rearing to alter paired-pulse responses suggests that the mechanisms mediating short-term plasticity appear unaffected by patterned sound deprivation. Lastly, the fact that LTP induction enhanced PPD indicates that, under* in vivo *conditions, LTP between MGN and A1 may involve presynaptic, release-related modifications at thalamocortical terminals. Future work is required to fully elucidate the role of these mechanisms in mediating long-term plasticity in thalamocortical sensory networks.

## Abbreviations

A1: Primary auditory cortex
ANOVA: Analysis of variance
fPSP: Field postsynaptic potential
i.p.: Intraperitoneal
ISI: Interstimulus interval
LTP: Long-term potentiation
MGN: Medial geniculate nucleus
PD: Postnatal day
PPD: Paired-pulse depression
PPF: Paired-pulse facilitation
PPR: Paired-pulse ratio
s.c.: Subcutaneous
SEM: Standard error of the mean
SPL: Sound pressure level
TBS: Theta-burst stimulation
WN: White noise.
